# Digital Applications for Videoterminal-Associated Dry Eye Disease

**DOI:** 10.3390/vision8040067

**Published:** 2024-11-28

**Authors:** Maria Angela Romeo, Giulia Coco, Andrea Taloni, Giovanna Carnovale-Scalzo, Vincenzo Scorcia, Giuseppe Giannaccare

**Affiliations:** 1 Department of Ophthalmology, University Magna Graecia of Catanzaro, 88100 Catanzaro, Italy; 2 Department of Clinical Sciences and Translational Medicine, University of Rome Tor Vergata, 00133 Rome, Italy; 3 Eye Clinic, Department of Surgical Sciences, University of Cagliari, 09124 Cagliari, Italy

**Keywords:** dry eye disease, digital solutions, mobile applications, health applications, ocular health, patient education, technology in healthcare, blinking dynamics, meibomian gland dysfunction, screen exposure

## Abstract

Dry eye disease (DED) has become increasingly prevalent in the digital era, largely due to prolonged screen exposure. The excessive use of digital devices contributes to inappropriate blink frequency and dynamics, leading to ocular surface dryness and discomfort. Additionally, digital screen use has broader implications for systemic health, including visual strain, headaches, and disrupted circadian rhythms caused by blue light exposure. Previous studies have shown that prolonged screen time correlates with altered blink frequency and increased symptom severity in DED patients, exacerbating the imbalance in tear film production and evaporation. Blinking dynamics, particularly blink rate and completeness, are crucial in maintaining ocular surface moisture. Incomplete blinking impairs meibomian gland function, reducing lipid secretion, which is essential for preventing tear evaporation. Raising patient awareness through educational material, ergonomic adjustments, and blinking exercises has been shown to mitigate these effects. Digital tools that provide targeted educational interventions can be particularly effective in improving blink dynamics and overall ocular comfort. This study evaluates the efficacy of digital applications in optimizing blinking dynamics and enhancing tear film stability. The findings suggest that these innovations improve patient outcomes by encouraging healthier eye care practices. However, further research is needed to assess their long-term impact across diverse populations.

## 1. Introduction

Over the last decade, there has been a marked increase in the use of digital devices in populations in developed countries across all age classes [1]. This shift towards increased screen time has introduced several health concerns, prominently including dry eye disease (DED). This is a multifactorial ocular condition characterized by disturbances in the tear film and ocular surface homeostasis, which may lead to symptoms such as ocular discomfort and reduced visual acuity [2]. The disease encompasses both quantitative and qualitative alterations in tear film production and stability, resulting in a complex interplay of contributing factors that impact visual function and overall quality of life [3]. As digital engagement has become increasingly embedded in professional and personal spheres, the need for targeted strategies to mitigate its impact on ocular health has become essential. Understanding and addressing the underlying causes of screen-induced visual discomfort is crucial for improving both physical well-being and workplace productivity, highlighting the urgent need for strategies to protect ocular surface health in the digital era [4]. The World Health Organization’s (WHO) global strategy on digital health of 2020–2025 underscores the critical role of digital tools in improving global healthcare access and quality. In this context, several types of software have been developed to manage DED in digital screen users. Digital applications, encompassing guided blinking exercises, and personalized recommendations for screen time regulation hold considerable potential for alleviating symptoms associated with DED by fostering effective patient collaboration. The integration of these innovative solutions into daily routines offers a promising approach to more comprehensive and effective management of DED in individuals subjected to extended periods of screen exposure.

This narrative review aims at elucidating the mechanisms through which visual screens exacerbate DED, exploring both physiological and behavioral aspects. Furthermore, it explores emerging digital solutions, particularly mobile applications, developed to offer therapeutic interventions and lifestyle modifications.

## 2. Methods

A literature search was conducted on PubMed in November 2024, using the query “dry eye” AND (“app*” OR “mobile” OR “software”). The references of the selected papers were inspected to search for additional articles. The iOS, Android, and Windows Stores were queried to find applications for the management of DED, using the keywords “eye”, “dry eye”, “blink”, “blink rate”, and “tear film”. Only applications in English were considered for further analysis.

## 3. Dry Eye Disease

DED is a multifactorial disease defined by quantitative and/or qualitative alterations in the tear film and ocular surface homeostasis [3]. Its pathogenesis involves a vicious cycle of tear film instability, hyperosmolarity, apoptosis of corneal/conjunctival cells, and inflammation of the ocular surface [5]. With a globally estimated prevalence ranging from 5.28% to 50% [6,7], DED poses a considerable socioeconomic burden [3].

Given its detrimental impact on quality of life, DED is one of the most frequent reasons for patients to seek ophthalmic care. DED often manifests with symptoms such as ocular discomfort, eye redness, dryness, foreign body sensation, itching, and photophobia [2,3]. In severe forms, conjunctival scarring and/or corneal complications may also occur.

DED is influenced by a broad spectrum of determinants, encompassing both non-modifiable and modifiable elements. Non-modifiable risk factors include age, female sex, and medical conditions like collagen vascular diseases, viral infections, and androgen insufficiency [8]. Modifiable risk factors, which are crucial in influencing both the onset and the exacerbation of the condition, include chronic use of topical medications, wearing contact lenses, vitamin deficiencies, postmenopausal estrogen therapy, and ocular surgical procedures, including, among others, cataract and refractive surgeries [9]. Additionally, environmental factors such as low humidity, air conditioning, wind exposure, smoke, and dust particles may accelerate tear evaporation and contribute to symptoms [10].

More recently, the extensive use of digital devices such as smartphones, tablets, televisions, and computers has been shown to significantly exacerbate DED [3,11]. Specifically, reduced blink rate, a common consequence of prolonged screen exposure, has been recognized as a critical factor in both the onset and worsening of DED among videoterminal users [12,13].

## 4. The Multifaced Impact of Digital Screen Use on Systemic Health

In recent years, a marked increase in the use of digital devices (e.g., smartphones, computers, laptops, and tablets) has been registered in developed countries across all age groups, with a particular surge in mobile media engagement. Before the coronavirus pandemic, people were already spending more time on online platforms compared to using printed media, specifically for an average of 27 extra minutes per day. This gap is anticipated to widen considerably, with digital platforms projected to lead by nearly 3 h by 2026 [1]. A recent study including data from England revealed that 68% of children regularly use a computer and 54% engage in electronic media by the age of 3 years [14]. This early exposure to digital devices underscores the growing influence of technology in modern life. Nonetheless, over the last decade, the progressive transition from in-person to remote work has brought about increased interference from technology in people’s lives. This trend underlines the deep integration of digital tools into both professional and personal spheres, highlighting the need for better awareness of their potential consequences on individuals’ health.

Health concerns related to the extensive use of visual displays are not limited to the eyes but also involve other structures of the body [11,15]. Specifically, musculoskeletal pain, particularly in the neck, back, and shoulders, is commonly reported, stemming from prolonged static postures and inadequate ergonomic setups during screen use. Furthermore, repetitive strain injuries such as carpal tunnel syndrome are prevalent among heavy computer users due to the sustained hand and wrist positioning [16]. Additionally, extended periods of sedentary behavior associated with screen use have been linked to increased risk of venous thromboembolism. Dermatologic conditions, including eczema, rosacea, and seborrheic dermatitis, also appear to be more frequent in cases of prolonged digital screen exposure, likely due to environmental factors such as dry air and increased artificial light exposure, which may increase skin sensitivity and exacerbate inflammation [11].

## 5. The Multifaced Impact of Digital Screen Use on Dry Eye Disease

The extensive use of digital devices has been associated with several visual disturbances. Symptoms can either be directly related to ocular surface stress, such as ocular discomfort, dryness, eye strain, epiphora, hyperemia, asthenopia, and temporary alterations in color perception, or be associated with the accommodative effort, including blurred vision, diplopia, and eye strain [17,18,19]. When investigating the potential link between visual display use and ocular surface dysfunctions, many authors have identified blinking dynamics as a major factor [20,21].

### 5.1. Blinking Dynamics

The physiological blinking rate has a range of 15–20 times per minute [22]. The spontaneous eye blink rate (sEBR) is modulated by both cognitive and task-related factors affecting the attentional load, such as mathematical or memory tasks [23,24], or by the emotional state [25,26]. In contrast to dialogues and conversations, which are accompanied by high levels of sEBR, working and reading, particularly if performed on digital screens, are characterized by low sEBR levels [22,27,28,29].

Several studies have reported that digital screen use is associated with reduced blink rates and completeness [4,30]. For instance, Patel et al. observed a reduction from a baseline value of 18.4 blinks/min to 3.6 blinks/min during computer use [29]. Similarly, Tsubota and Nakamori reported office workers to exhibit a blink rate of 22 blinks/min in relaxed conditions, which decreased to 7 blinks/min when engaged in digital screen use [22,31].

### 5.2. Blinking and Dry Eye

A reduced blink rate and completeness can have an impact on DED. The Osaka cross-sectional study, which recruited 561 patients aged 22–65 years with a mean videoterminal use of 7.9 h/day, reported an increased incidence of DED signs, as shown by alterations in both the Schirmer test and tear breakup time (TBUT) and the presence of ocular surface epithelial damage [4].

Interestingly, Moon JH et al. showed that a 4-week cessation of smartphone use in children aged 7–12 years with DED led to substantial improvements in non-invasive tear breakup time (NIBUT), superficial punctate epitheliopathy, and Ocular Surface Disease Index (OSDI) scores. By the end of the smartphone use-free period, all children were no longer categorized as having DED [32].

### 5.3. Blinking and Meibomian Gland Dysfunction

The correlation between blinking dynamics and meibomian gland dysfunction (MGD) plays a critical role in the pathogenesis of DED [33]. Meibomian glands secrete meibum, a lipid substance which serves as a barrier to reduce tear evaporation [34,35]. Normal blinking behavior ensures the consistent delivery and even distribution of meibum across the ocular surface [36,37]. However, altered blinking dynamics, such as incomplete blinks and a reduced blink frequency, can significantly disrupt this process, leading to a reduction in meibum release with consequent thinning of the lipid layer, increased tear film instability, and osmolarity, which in turn lead to increased tear evaporation and exacerbation of DED symptoms [34,36,37,38]. As a matter of fact, incomplete or infrequent blinking has been associated with meibomian gland dropout and a decline in lipid secretion [39]. Studies also demonstrated that individuals exhibiting a higher ratio of incomplete blinks had significantly reduced TBUT and high OSDI scores, indicating a link between blink dysfunction and MGD progression [34].

Further, a compromised lipid layer due to both abnormal blinking and gland dysfunction leads to compensatory reflex aqueous tear secretion, which may paradoxically worsen blink patterns and tear film stability, perpetuating the cycle of DED [40,41].

Additionally, bacterial activity significantly contributes to MGD by producing lipolytic enzymes that degrade meibum, converting neutral lipids into free fatty acids [42]. This condition is often exacerbated by incomplete blinks, as insufficient meibum expression allows bacteria and their byproducts to degrade the lipid layer, leading to chronic inflammation and tear film disruption [33].

### 5.4. Blue Light Exposure

The increasing use of digital screens, including smartphones and computers, has raised concerns about the effects of blue light on eye health. The literature suggests that the blue light emitted by these devices contributes to visual discomfort and can generate reactive oxygen species (ROS), thus reducing cellular viability in the corneal epithelium in vitro models [43]. In addition to contributing to DED, the blue light emitted by digital screens can disrupt circadian rhythms and increase eye strain. As a matter of fact, prolonged exposure to blue light from devices, especially in the evening, may impair sleep quality due to its effect on melatonin suppression [44].

## 6. Targeted Approaches for Dry Eye Disease in Digital Screen Users

### 6.1. Patient Education

Given the mounting evidence regarding the detrimental effects of extended visual display terminal usage, the most straightforward solution, although often impractical, would be a reduction in extended screen time. However, as these technologies are embedded in daily life, it is essential to focus on educating patients about their safe and effective use [7,45]. This involves encouraging responsible screen time management and routine practices to mitigate the potential ocular and systemic risks associated with sustained digital engagement.

#### 6.1.1. Awareness

Today, many digital applications include features for symptom evaluation and educational materials about DED, contributing to patient awareness [46,47]. It is important to emphasize the need for early DED diagnosis, which can prevent the onset of a vicious cycle of worsening symptoms that become harder to manage over time. A key component in addressing these issues is patient education, emphasizing the need for responsible screen time management and awareness of the risks associated with prolonged exposure. Education should focus on several key aspects: blinking exercises, ergonomic adjustments, and frequent breaks to reduce eye strain and promote ocular health.

#### 6.1.2. Ergonomic Adjustments

Patients should be instructed to maintain correct body posture and optimize their workstations. This includes positioning the computer screen 15 to 20 degrees below eye level and 20 to 28 inches from the eyes, ensuring proper lighting to avoid glare, as well as using anti-glare screens or filters when necessary [48]. Additionally, chairs should be comfortably padded and adjusted so that the feet rest flat on the floor, with arms supported during typing and wrists not resting on the keyboard [48].

#### 6.1.3. Take Regular Breaks

Taking regular breaks is another crucial preventive measure. To minimize eyestrain, patients should be encouraged to rest their eyes for 15 min after every 2 h of continuous computer use and follow the 20–20–20 rule: every 20 min, look at something 20 feet away for 20 s.

#### 6.1.4. Blink Frequently

Frequent blinking should also be practiced, keeping the eyes moist to prevent dry eye symptoms [49]. As digital devices are now integrated into daily life, educating users about safe practices becomes crucial for reducing DED symptoms and improving long-term eye health. This includes understanding the importance of ergonomics, proper posture, and breaks, which play a critical role in preventing computer vision syndrome and reducing the discomfort associated with prolonged screen exposure.

### 6.2. Blinking Exercises

Research has emphasized that preventative strategies for DED could include deliberate blinking exercises. Voluntarily increasing blink frequency has been shown to enhance the secretion of meibum from the meibomian glands, improving the lipid layer’s distribution on the ocular surface, which in turn bolsters tear film stability [34,38]. This is particularly effective in mitigating blink suppression, a common consequence of prolonged screen exposure, where blink rates can drop substantially.

In this context, various innovative devices and types of software have been investigated, aimed at increasing both the frequency and quality of blinking. Several types of visual stimuli, such as screen blurring, light flashes, flashing borders, and pop-up animations, have been assessed to determine the optimal method for increasing blink frequency without compromising user comfort [50]. Among these options, screen blurring has been found to be the most effective, leading to a significant increase in blink rates while remaining the least intrusive and most satisfactory for users [46,50]. Moreover, liquid crystal technology devices like wearable Wink Glasses (WGs) become opaque after five seconds of non-blinking and return to transparency when a blink is detected, demonstrating that users of WGs experience a significant increase in blink rate, as well as stabilized TBUT, after reading sessions [51]. Collectively, these approaches target the two primary contributors to DED: inadequate tear film replenishment and excessive tear evaporation.

### 6.3. Modulation of Blue Light Exposure

Studies have demonstrated that reducing blue light exposure can improve subjective visual comfort in users who engage in prolonged screen use [44]. In this context, modern digital devices come equipped with advanced screen adjustment features designed to alleviate visual fatigue and enhance user comfort. One notable feature is the “Night Shift” or similar blue light reduction modes, which are available on many operating systems. This functionality adjusts the color temperature of the screen to reduce blue light exposure, especially during evening hours.

## 7. Emerging Digital Solutions for Dry Eye Disease

The first-line approach in DED management should be focused on patient education. Among videoterminal users, optimizing the location of the visual display, implementing lifestyle adjustments, incorporating blink training exercises, and utilizing humidifiers in the workstation environment are all strategies to consider.

Taking inspiration from the progress of digital solutions in general healthcare, where promising applications have been shown to be effective tools for enhancing treatment adherence in adults with chronic illnesses compared to traditional care methods [45,52], similar mobile applications have been developed to help patients with DED. The importance of new technologies has also been highlighted in the WHO global strategy on digital health 2020–2025 [53]. The FDA distinguishes between wellness and health applications. Wellness applications are designed to promote general well-being and healthy lifestyles but do not diagnose, treat, or prevent diseases. Examples include apps that track physical activity, provide nutritional advice, or promote relaxation and meditation. These do not require FDA approval as they are not classified as medical devices. In contrast, health applications are classified as mobile software designed to diagnose, track, or treat medical conditions. As of July 2018, the FDA had approved over 300 health applications [54,55], making them part of regulated medical software aimed at enhancing patient outcomes. Overall, several health applications have emerged in the field of ophthalmology, covering various aspects such as glaucoma, visual acuity assessment, dry eye management, and patient education. Digital health applications have been designed to offer personalized, non-invasive strategies to manage DED. These aim to modulate blinking behavior, to instruct on correct daily eye care, and to reinforce adherence to pharmacological treatments (Table 1).

Among the applications focused on blink stimulation, “DryEyeRhythm” (Version 5.0.2; Juntendo University, Bunkyo City, Tokyo, Japan) stands out by offering a comprehensive approach. This app utilizes smartphone cameras to monitor blinking characteristics, assess tear film stability, and provide detailed, personalized feedback based on users’ ocular health metrics. It also integrates the OSDI questionnaire to evaluate symptoms. The app enables users to complete a quick DED assessment, generating a “dry eye score” based on blink data and lifestyle factors such as stress and sleep patterns. Additionally, the app provides visual insights into the user’s condition through a “Dry Eye Map” and displays symptom trends on a graph and calendar.

Moreover, anonymized data are collected by Juntendo University for ongoing research. Interestingly, DryEyeRhythm is the only mobile app for DED that has been evaluated in the scientific literature. As a matter of fact, the app’s diagnostic capabilities have been validated in a prospective, cross-sectional study, which demonstrated strong internal consistency, a positive correlation with clinical measures, and high discriminant validity. The results revealed that DryEyeRhythm offered high positive and negative predictive values (91.3% and 69.1%, respectively) and a Receiver Operating Characteristic (ROC) curve value of 0.910, indicating its reliability and validity in assessing DED. This study also confirmed the app’s discriminant validity, where app-based assessments accurately differentiated between participants with and without DED. For DED diagnosis, the app showed a high positive predictive value (91.3%) and a respectable negative predictive value (69.1%) [47]. These results further underscore the app’s reliability in offering a valid, non-invasive diagnostic tool for DED, enhancing its clinical utility in both screening and ongoing management.

Another notable application is “DryiRelief” (Version 1.14; Optometrist Calgary INC, Calgary, AB, Canada), which offers a range of functionalities, including specific eye care exercises, warm compresses, eyelid hygiene through lid wipes, eye drop instillation tracking, and reminders to blink. This app provides a multifaceted approach to managing dry eye symptoms by supporting various aspects of ocular care. The app is organized into three main sections with an intuitive interface: OSDI questionnaires, exercises/activities, and auditory reminders. All activities are summarized in the “Workout Scores” section, allowing users to track their progress over time. Among these exercises, the warm compresses feature includes a 10 min timer to ensure proper application duration, while the lid wipe section guides the user through a 2 min cleansing routine. The app also tracks eye drop instillations and encourages the use of omega-3 supplements. Additionally, the app introduces the “20–20–20 rule” to help reduce eye strain, featuring an English language introductory video that explains the rule’s benefits before the start of the timer. This rule encourages users to take a 20 s break every 20 min, focusing on an object 20 feet away, promoting eye relaxation during extended screen use [48]. The blinking exercises are supported by the phone’s camera, which counts blinking episodes and provides real-time feedback to ensure proper performance. Beyond these core features, DryiRelief also offers additional functions such as a blink counter, educational training videos, and a dry eye product store. Users can also access recommendations on advanced treatment options and educational resources like the TFOS DEWS II Report Executive Summary (2017) [7], which provides detailed information on the treatment of dry eye disease.

“Eye Care Plus” (Version 3.3.8; Uptodown, Málaga, Spain) also provides a diverse set of tools, including eye exercises, vision tests, and reminders for breaks and blinking to reduce eye strain. Users can earn points through daily vision exercises and tests, which can be scheduled for an individualized experience.

The app also aims to raise awareness about eye health, emphasizing proper nutrition and care. As a matter of fact, it offers updates on medical articles alongside practical eye health and nutrition tips. Additionally, patients can submit questions and access basic first aid guidance for eye emergencies. Overall, the app features easy navigation and provides detailed instructions for each exercise. A progress chart allows users to monitor their improvements over time, and the app’s modern, youthful interface makes it engaging and easy to use.

“EyeBlink App” (Version 3.7.4; Andrej Fogelton, Ph.D., Slovakia) provides specific eye exercises and regular reminders to blink at appropriate intervals, aiding in maintaining ocular hydration. Remarkably, the app features a blink detector that uses the camera to monitor blink rate in real time. Reminders only disappear after a blink is detected, employing advanced AI technology. To work effectively, the user must keep their face visible within the camera frame, which requires having their phone readily available.

“MyDryEye” (Version 1.02.2; Wolffsohn Research Ltd., Lisburn, County Antrim, Ulster) offers a comprehensive and user-friendly platform designed to assist in managing dry eye symptoms and treatments. It provides a structured approach to tracking symptoms such as redness, itchiness, burning sensations, and blurred vision, along with their severity and duration. Users can also monitor various treatments, including artificial tears, warm compresses, and medications, evaluating their effectiveness and adherence. The app enhances treatment consistency by sending customizable reminders to help users stay on track with their medication and other therapies. Additionally, it features an integrated directory that allows the user to find local ophthalmologists and optometrists, facilitating access to specialized care.

“Blink Break” (Version 3.0; Johnson & Johnson Surgical Vision, Inc. 2021, Singapore) is designed to educate patients on the importance of blinking to maintain ocular lubrication and protection. The app offers training exercises to improve blinking habits and allows users to set blinking reminders based on their doctor’s recommendations, with options for intervals of 30 min, 1 h, or 2 h.

“Eye Buddy” (Version 2.0; EyeBuddy, Santa Ana, IL, USA) is an innovative app designed to help users maintain and improve their eye health through personalized exercises and education. It offers a range of eye care routines, including blinking exercises, focus training, and relaxation techniques, to alleviate eye strain. Users can set reminders to take regular breaks and track their progress over time. An engaging feature of EyeBuddy is the “Level Up and Workout Worldwide”, where users collect rewards as proof of their efforts. This approach allows users to showcase their achievements on a global platform and engage in eye workouts alongside others around the world. Additionally, the app includes educational content about eye health.

## 8. Conclusions

Digital applications designed to promote effective blinking behavior offer significant potential for mitigating DED symptoms associated with prolonged screen use. Also, by integrating these digital tools into daily routines, patients can be educated in optimizing screen settings to reduce visual strain, improving tear film stability and overall ocular comfort. Moreover, these apps enhance accessibility, convenience, and clarity of healthcare for patients while simultaneously offering doctors extensive technical and advisory support. This dual advantage ultimately elevates the quality of medical care. Therapeutic success depends on effective clinician–patient interactions and their shared responsibility for outcomes. Using mobile technologies in clinical practice can enhance these interactions, making them more effective, and increasing adherence to treatment. Despite the growing interest in digital applications, there are still relatively few studies that rigorously evaluate their benefits in a clinical setting. Further investigation into the long-term efficacy and applicability of digital tools in diverse patient populations is required to meet the evolving needs of patients and improve outcomes in the management of DED.

## Figures and Tables

**Table 1:** Digital applications for dry eye disease in individuals subjected to prolonged screen exposure.

App Name	Developer	System Requirements	Launch Date	Blinking Exercises	Blinking Monitoring	Pop-Up Reminders	Other Functionalities	Progresses Tracking
**DryEyeRhythm** (Version 5.0.2)	Juntendo University, Bunkyo City, Tokyo, Japan	iOS/Android	2022	No.	Yes: blink checks via the camera.	No.	Lifestyle input (e.g., stress, sleep pattern) in correlation with DED Questionnaires for DED.	Yes. Visual progress chart.
**DryiRelief** (Version 1.14)	Optometrist Calgary INC, Calgary, Alberta, Canada	iOS	2019	Yes.	Yes: camera counts blink episodes and monitors blinking rate during exercises.	Yes: auditory and visual reminders for eye care exercises (e.g., blinking, warm compresses and eye drop usage).	Hot compress timer and guided eyelid hygiene routines. Eye drop instillation tracking. Omega-3 supplement reminders. Educational resources: TFOS DEWS II Report Executive Summary (2017) and “20-20-20 rule”. Access to dry eye product recommendations and a store.	Yes: “Workout Scores” section.
**EyeCarePlus** (Version 3.3.8)	Uptodown, Málaga, Spain	Android	2023	Yes.	No real-time blinking monitoring.	Yes: reminders for taking breaks from the screen, blinking reminders, customizable eye exercises reminders.	Vision tests (e.g., Snellen chart, astigmatism test). Eye exercises for improving vision and reducing eye strain. Educational content on eye health (e.g., articles, tips, and healthy recipes). First aid guidance for eye emergencies.	Yes: Progress charts.
**EyeBlink** (Version 3.7.4)	Andrej Fogelton, Ph.D. Slovakia	Windows	2024	Yes (requires face visibility within the camera frame for accurate monitoring).	Yes: camera-based blink detector to monitor blink rate in real time.	Yes: reminders to blink at proper intervals, (reminders only dismiss once the app detects a blink).	No.	No.
**MyDryEye** (Version 1.02.2)	Wolffsohn Research Ltd., Lisburn, County Antrim, Ulster.	iOS/Android	2023	No.	No blinking monitoring.	Yes: treatment schedules, customizable reminders (eye drops and warm compresses).	Symptom Tracker: logs symptoms like redness, burning, and blurred vision. Treatment adherence (e.g., artificial tears and other therapies). Expert Finder: provides a directory to locate local dry eye specialists.	Yes: tracks symptom progression over time, including monitoring the effectiveness of treatments.
**BlinkBreak** (Version 3.0)	Johnson & Johnson Surgical Vision, Inc. 2021, Singapore	iOS/Android	2021	Yes.	No blinking monitoring.	Yes: personalized blinking. Reminders based on their doctor’s recommendation (with intervals of 30 min, 1 h, or 2 h).	Educational content. Reminders to blink at regular intervals.	No.
**EyeBuddy** (Version 2.0)	EyeBuddy, Santa Ana, IL, USA	iOS/Android	2021	Yes: personalized blinking exercises and focus training.	No.	Yes: Screen breaks reminders.	Focus training and relaxation techniques for reducing eye strain. Educational content “Level Up and Workout Worldwide”, where users earn titles and rewards for progress and compete with others.	Yes.

## Data Availability

No new data were created or analyzed in this study. Data sharing is not applicable to this article.
